# The Effect of Dietary Fibre on Gut Microbiota, Lipid Profile, and Inflammatory Markers in Patients with Type 2 Diabetes: A Systematic Review and Meta-Analysis of Randomised Controlled Trials

**DOI:** 10.3390/nu13061805

**Published:** 2021-05-26

**Authors:** Omorogieva Ojo, Osarhumwese Osaretin Ojo, Nazanin Zand, Xiaohua Wang

**Affiliations:** 1 School of Health Sciences, University of Greenwich, Avery Hill Campus, Avery Hill Road, London SE9 2UG, UK; 2 South London and Maudsley NHS Foundation Trust, University Hospital, Lewisham High Street, London SE13 6LH, UK; Osarhumwese.Ojo@slam.nhs.uk; 3 School of Science, University of Greenwich, Medway Campus, Chatham ME4 4TB, UK; N.ZandFard@greenwich.ac.uk; 4 The School of Nursing, Soochow University, Suzhou 215006, China; wangxiaohua@suda.edu.cn

**Keywords:** type 2 diabetes, lipid profile, gut microbiota, inflammatory markers, body mass index, lipopolysaccharide, dietary fibre

## Abstract

Background: A disequilibrium of the gut microbial community has been closely associated with systemic inflammation and metabolic syndromes including type 2 diabetes. While low fibre and high fat diets may lead to dysbiosis of the gut microbiome as a result of the loss of useful microbes, it has been reported that a high fibre diet may prevent the fermentation of protein and may promote eubiosis of gut microbiota. Aim: This review aims to evaluate the effect of dietary fibre (DF) on gut microbiota, lipid profile, and inflammatory markers in patients with type 2 diabetes. Methods: The PRISMA framework was relied on to conduct this systematic review and meta-analysis. Searches were carried out using electronic databases and reference list of articles. Results: Eleven studies were included in the systematic review, while ten studies were included in the meta-analysis. The findings revealed five distinct areas including the effects of DF on (a) gut microbiota (122 participants); (b) lipopolysaccharides (LPS, 79 participants) and lipopolysaccharides binding protein (LBP, 81 participants); (c) lipid profile; (d) inflammatory markers; and (e) body mass index (BMI, 319 participants). The relative abundance of *Bifidobacterium* increased by 0.73 (95% CI: 0.57, 0.89) in the DF group in contrast to the control (*p* < 0.05). With respect to LPS, the level was lower in the DF group than the control and the difference was significant (*p* < 0.05). The standardised mean difference for LPS was −0.45 (95% CI: −0.90, −0.01) although the difference between the two groups in relation to LBP was not significant (*p* = 0.08) and the mean difference was 0.92 (95% CI: −0.12, 1.95). While there was a decrease of −1.05 (95% CI: −2.07, −0.02) with respect to total cholesterol (356 participants) in the DF group as compared with the control (*p* < 0.05), both groups were not significantly different (*p* > 0.05) in the other lipid parameters. The difference between the groups was significant (*p* < 0.05) in relation to C-reactive protein, and the mean difference was 0.43 (95% CI: 0.02, 0.84). This could be due to the short duration of the included studies and differences in participants’ diets including the amount of dietary fibre supplements. However, the groups were not significantly different (*p* > 0.05) with respect to the other inflammatory markers. The meta-analysis of the BMI showed that the DF group decreased by −0.57 (95% CI: −1.02, −0.12) as compared with the control and this was significant (*p* < 0.01). Conclusion: DF significantly (*p* < 0.05) increased the relative abundance of *Bifidobacterium* and significantly decreased (*p* < 0.05) LPS, total cholesterol, and BMI as compared with the control. However, DF did not seem to have an effect that was significant on LBP, triglyceride, HDL cholesterol, LDL cholesterol, IL-6, TNF-α, adiponectin, and leptin. These findings have implications for public health in relation to the use of dietary fibre in nutritional interventions and as strategies for managing type 2 diabetes.

## 1. Introduction

A disequilibrium of the gut microbial community has been closely associated with systemic inflammation and metabolic syndromes [1,2]. There is evidence that dysbiosis of the gut microbiome has an effect on the pathogenesis of type 2 diabetes as it regulates inflammatory markers, interacts with constituents of the diet, modulates the permeability of the gut, as well as glucose and lipid metabolism, sensitivity of insulin, and balance of energy [3,4,5]. The disruption of microbial eubiosis could be due to the quality of the diets, especially high fat diets and those that are high in sugar and low in dietary fibre [6]. For example, Western-style diets, which are low in dietary fibre and other microbiota-accessible carbohydrates, may contribute to a reduction in microbial diversity and could lead to the depletion of specific bacterial taxa in the digestive ecosystem [6,7]. This process may cause microbial dysbiosis or changes in the profile of gut microbiota which could impair the integrity of the wall of the intestine and cause gut permeability, thus, enabling the translocation of toxins from the gut lumen to the systemic circulation [4]. The fermentation of dietary fibres by gut bacteria produces primarily short-chain fatty acids (SCFAs) including propionic, butyric, and acetic acids, hence, dysbiosis may lead to significant differences in the concentration of SCFAs in the intestines, of which a deficiency has been associated with type 2 diabetes [8,9]. The composition of the gut microbiota and interactions between the different species of microbes influence the type and amount of SCFAs including butyric, acetic, and propionic acid. In this regard, a low intake of dietary fibre may cause reduced production of SCFAs and lead to the utilisation of substrates that are less favourable, such as proteins and fat, by the gut microbiome and the production of potentially detrimental metabolites including lipopolysaccharide (LPS), a metabolic endotoxemia often associated with microbiota dysbiosis [6,10,11].

An increase in the permeability of the mucosa wall of the intestine and the movement of LPS through the epithelium can elicit inflammation and lead to insulin resistance and development of type 2 diabetes [10]. In particular, the endotoxemia cN cause low-grade inflammation and oxidative stress which may cause insulin resistance, beta cell dysfunction, hyperglycemia, hyperlipidemia, and obesity [4]. A high level of endotoxemia has been found to increase the concentrations of tumour necrosis factor α (TNF-α) and interleukin 6 (IL-6) and the promotion of insulin resistance [12].

Although some gut microbes and their products such as lipopolysaccharides promote metabolic endotoxemia and low-grade inflammation, others including *Roseburia intestinalis*, *Bacteroides fragilis*, *Akkermansia muciniphila,* and *Lactobacillus plantarum* may contribute to the improvement of glucose metabolism by stimulating anti-inflammatory cytokines and chemokines [3]. The effect of type 2 diabetes can be profound in terms of its acute and chronic complications and with significant costs to health services around the world.

### 1.1. Description of the Intervention

Dietary fibres are carbohydrate polymers that are not digested or absorbed in the small intestine and are usually fermented in the colon resulting in the production of SCFAs, some of which may be used as sources of energy [13].

Dietary fibres include non-starch polysaccharides (NSP), which are constituents of plant cell wall and include cellulose, hemicelluloses, pectins, gums, mucilages, and beta-glucans, as well as other components such as lignin which are related to carbohydrates that are non-digestible in cell walls of plants [13]. NSP can be further divided into (a) soluble fibres, such as those from oats, psyllium, pectin, and guar gum, which can have an impact on glucose and lipid absorption [14] and (b) insoluble fibres, which are slowly or not completely fermented in the large intestine and have a significant effect on bowel habits [13]. Furthermore, non-digestible oligosaccharides including fructo-oligosaccharides and gluco-oligosaccharides have been shown to influence abundance and diversity of gut microbiota [14].

### 1.2. How the Intervention Might Work

Aliasgharzadeh et al. [5] demonstrated in their study that an imbalance in the intestinal microbiota was a risk factor for type 2 diabetes. This was based on the understanding that changes in the ratio of gut microbiota can cause loss of integrity of the intestinal mucosal barrier and lead to bacteria translocation [10]. In addition, the disruption of intestinal barrier increases intestinal mucosal permeability, allowing the translocation of LPS, a metabolic endotoxemia [10]. Thus, microbial dysbiosis could cause an increase in inflammatory activation via promotion of an immune response to LPS and this may contribute to insulin resistance and type 2 diabetes [10]. The role of NSP in human health has been demonstrated by its improvement in the composition and metabolic products of the gut microbiota, including increasing the abundance of health-promoting bacteria and the production of SCFAs [15,16]. The SCFAs produced by the fermentation of polysaccharides have been shown to have a significant effect in improving glycometabolism-related diseases [15,17]. Furthermore, it has been suggested that the modulation of specific bacteria that is related to the dysregulation of glucose metabolism by dietary fibre may be an effective method of promoting glucose homeostasis and improving lipid profile [15,18,19].

### 1.3. Why It Is Important to do This Review

In our previous review [20], we found that dietary fibre significantly improved the relative abundance of *Bifidobacterium*, total SCFAs, and glycated haemoglobin. However, the findings did not demonstrate any significant impact of dietary fibre on fasting blood glucose, homeostatic model assessment of insulin resistance, acetate, propionate, butyrate, and adverse events. Therefore, the current review is a follow-up to the previous review and focuses on the role of dietary fibre on lipid profile and inflammatory parameters. Our understanding of gut microbiome will no doubt promote the study of dietary fibre and its effect on human health and nutrition [21,22]. In addition, new knowledge on the modulation of gut microbiota by dietary fibre is essential for developing effective strategies to improve human health and to manag microbiota-related diseases [21]. This is particularly significant as most microbiome-associated pathologies such as type 2 diabetes are increasing globally [6,8]. While there is evidence that a high fat diet may be involved in the disruption of gut microbiota symbiosis resulting from the loss of useful microbes [6,23,24], it has also been revealed that a high fibre diet may prevent the fermentation of protein and promote gut microbial eubiosis [6].

A complete understanding of how the communities of microbes and specific bacteria cause, respond to, or contribute to diseases such as type 2 diabetes is not fully understood and continues to evolve [9]. On the one hand, according to Mitchell et al. [11], an increased level of lipopolysaccharide, which is associated with microbiota dysbiosis, has been implicated in conditions such as type 2 diabetes and cardiovascular disease. On the other hand, Ebrahimzadeh Leylabadlo et al. [8] noted that the effect of lipopolysaccharide concentrations on glucose and lipid metabolism in humans has not been studied extensively. Furthermore, reviews conducted previously have focused only on the effect of whole diet and/or lifestyle interventions [25] or probiotics [8] on gut microbiota in patients with type 2 diabetes.

### 1.4. Aim

The aim of this review is to examine the effect of dietary fibre on gut microbiota, lipid profile, and inflammatory markers in patients with type 2 diabetes.

## 2. Methods

The preferred reporting items for systematic review and meta-Analysis (PRISMA) [26] was the framework used for this review.

### 2.1. Types of Studies

The studies included were randomised controlled trials.

### 2.2. Types of Participants

People with type 2 diabetes were the participants included in this review.

### 2.3. Types of Interventions

Dietary fibre including microbiotic diet was the intervention.

### 2.4. Types of Outcome Measures

The outcome measures of interest included the following:

- The relative abundance of gut microbiota (genera only) (*Bifidobacterium);*
- Lipopolysaccharides and lipopolysaccharides binding protein;
- Lipid profile, i.e., high density lipoprotein (HDL) cholesterol, total cholesterol, low density lipoprotein (LDL) cholesterol, triglycerides;
- Inflammatory markers, i.e., high sensitivity C-reactive protein (hsCRP), interleukin 6 (IL-6), tumour necrosis factor α (TNF-α), adeponectin, and leptin;
- Body mass index (BMI).

### 2.5. Search Methods for Identification of Studies

Electronic databases including EBSCO-host that includes Health Sciences Research Databases (encompassing MEDLINE, Academic Search Premier, APA PsycInfo, Psychology and Behavioral Sciences Collection, APA PsycArticles databases, and CINAHL Plus with Full Text), EMBASE, Google Scholar, and the reference lists of articles were searched for relevant articles. A population, intervention, outcome and study (PICOS) design was the framework used for the searches which were conducted from database inception to 3 March 2021 (Table 1). Synonyms and medical subject headings (MesH) were used as search terms, and these were combined using Boolean operators (OR/AND). Two researchers (O.O. and O.O.O.) independently conducted the searches and were cross-checked by N.Z. and X.W. Differences were resolved through discussion and consensus. Records of searches from databases were transferred to EndNote (Analytics, Philadelphia, PA, USA) and the duplicates were removed.

### 2.6. Data Collection and Analysis

#### 2.6.1. Selection of Studies

**Figure 1:**
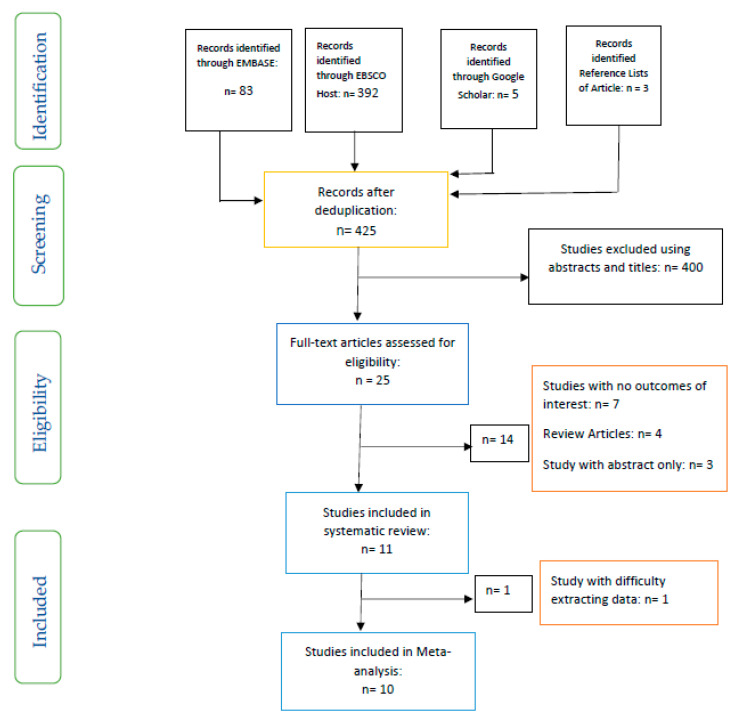
PRISMA flow chart of studies included.

Inclusion and exclusion criteria were used to select the studies included and those excluded based on a PRISMA flow chart (Figure 1).

The inclusion criteria included the following: Only randomised controlled studies involving patients with type 2 diabetes were included in this review. Studies with participants aged 18 years or over and with outcomes involving gut microbiota, lipopolysaccharide, lipid profiles, inflammatory parameters, and anthropometric measurements were also included in this review.

The exclusion criteria included the following: Studies involving patients with type 1 diabetes, prediabetes, or gestational diabetes, and participants aged below 18 years were excluded from this review. Animal studies and those involving probiotics were also excluded.

#### 2.6.2. Data Extraction and Management

One researcher (O.O.) extracted the data from the selected articles, which was cross-checked by the other three researchers (O.O.O., N.Z., and X.W.). Changes from baseline and final values in the intervention group were compared to the control. Web Plot Digitizer [27] was used to extract data from graphs in the Medina-Vera et al. [28] and Pedersen et al. [29] studies. The units of measurements for lipid parameters were converted to mmol/L, while median and 1st and 3rd quartiles were converted to means and standard deviations, respectively, using an Excel table for estimating mean and standard deviation from median and quartiles.

#### 2.6.3. Assessment of Risk of Bias in Included Studies

Two researchers (O.O. and O.O.O.) used the domain-based risk assessment tool [30] to evaluate the risk of bias of the studies included by and this was cross-checked by the two other researchers (N.Z. and X.W.). The domains assessed were allocation concealment, the random sequence generation, blinding of outcome assessment, blinding of participants and personnel, selective reporting, incomplete outcome data, and other biases [30]. The Review Manager 5.3 software (Copenhagen, Denmark) [31] was used to carry out the risk assessment. Furthermore, the quality of the evidence was assessed by means of the Critical Appraisal Skills Programme [32] checklist for randomised controlled trials.

#### 2.6.4. Data Analysis

The Review Manager (RevMan) 5.3 software [31] was used to carry out the meta-analysis. One study at a time was removed from the meta-analysis to facilitate the sensitivity analysis, and thus determine the level of consistency of the results. Heterogeneity of studies was measured by *I*^2^ statistic [30] and expressed as percentage. The random effects model was used for meta-analysis in data with high heterogeneity, while the fixed effects model was used in data with low to medium heterogeneity. For lipopolysaccharides, the standardised mean difference was used for the analysis due to differences in the units of measurements of the included studies. Furthermore, both change scores (change from baseline) were combined with final scores in the meta-analysis [30].

#### 2.6.5. Effect Size

The results of the meta-analysis are presented as forest plots, while the overall effect of the intervention was based on *p* < 0.05 with respect to statistical significance.

## 3. Results

There were eleven studies included in the systematic review, while the meta-analysis had ten studies (Figure 1). The type of study, details of sample size, the mean age/range, the aims of studies, interventions, and results of the included studies are outlined in Table 2. While one study each was conducted in Belgium [33], Canada [34], China [35], Japan [36], Mexico [28], Norway [37], and UK [29], four studies were carried out in Italy [38,39,40,41]. All the studies included were randomised controlled trials.

### Risk of Bias of Included Studies

**Figure 2:**
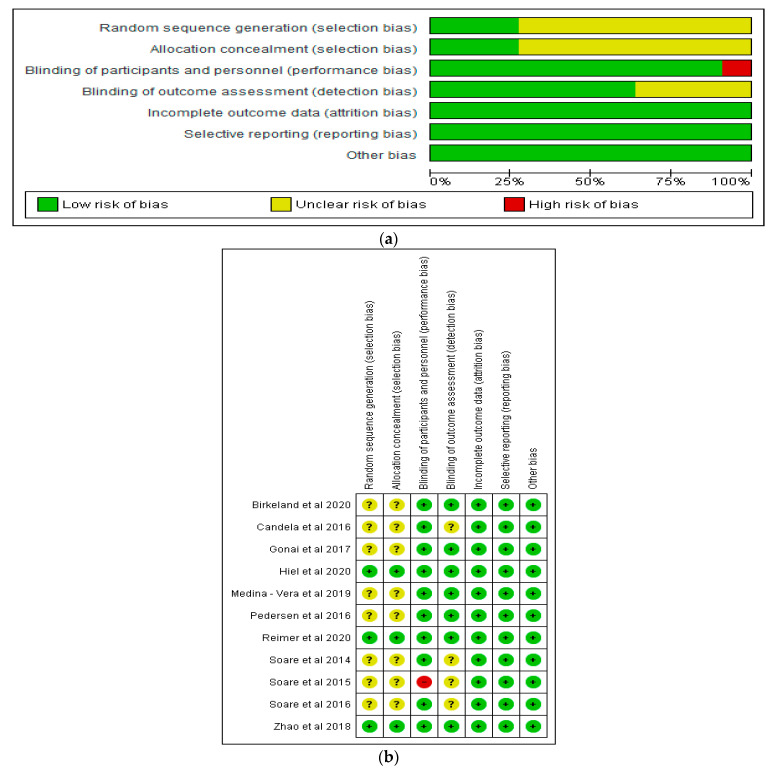
(**a**) Graph of risk of bias of included studies; (**b**) summary of risk of bias of included studies.

With respect to incomplete outcome data and selective reporting, all the studies demonstrated a low risk of bias (Figure 2a,b). However, some studies showed an unclear risk of bias in relation to random sequence generation, allocation concealment, and blinding of outcome assessment, while one study [40] showed a high risk of bias in relation to blinding of participants and personnel (Figure 2a,b).

The findings of the current systematic review (Table 2) and meta-analysis identified five distinct areas including the effects of dietary fibre on (a) gut microbiota (122 participants); (b) lipopolysaccharides (79 participants) and lipopolysaccharides binding protein (81 participants); (c) lipid profile, (d) inflammatory markers, and (e) body mass index (319 participants).

(a) Gut Microbiota

**Figure 3:**
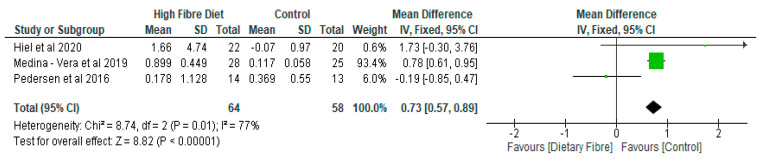
The effect of dietary fibre on Bifidobacterium (%).

Medina-Vera et al. [28] found that the consumption of a diet rich in fibre increased the levels of *Faecalibacterium prausnitzii* and *Akkermansia muciniphil* which are two bacterial species that have been known to have anti-inflammatory effects. The high fibre diet also promoted the abundance of *Bifidobactrium longum*. Furthermore, Reimer et al. [34] found that there was a significant increase in the dietary fibre group with respect to the relative abundance of *Collinsella, Parabacteroides,* and *Roseburia* as compared with the control. The effects of dietary fibre on gut microbiota have previously been described in our earlier review [20]. In the current meta-analysis of the relative abundance of *Bifidobacterium*, there was an increase of 0.73 (95% CI: 0.57, 0.89) in the dietary fibre group as compared with the control (*p* < 0.05) (Figure 3).

(b) Lipopolysaccharide (LPS) and Lipopolysaccharide Binding Protein (LBP)

**Figure 4:**
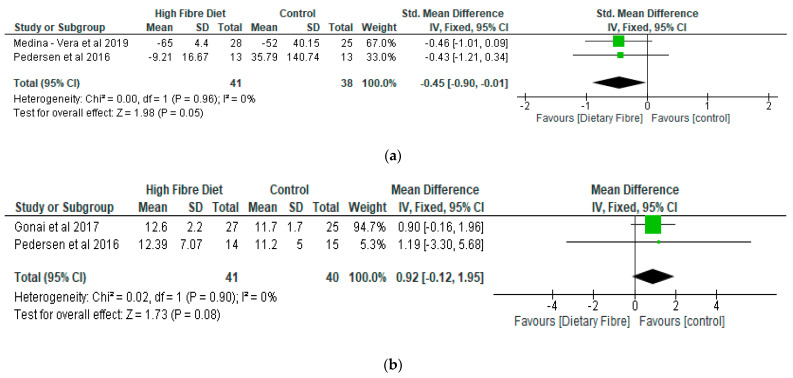
The effect of dietary fibre on (**a**) lipopolysaccharide (standardised mean difference); (**b**) lipopolysaccharide binding protein (µg/mL).

With respect to LPS, the meta-analysis showed that there was a significantly lower level of lipopolysaccharide (*p* < 0.05) in the dietary fibre group as compared with the control, with a standardised mean reduction of −0.45 (95% CI: −0.90, −0.01) (Figure 4a). However, there was no significant difference (*p* = 0.08) between the dietary fibre group as compared with the control in relation to LBP with a mean difference of 0.92 (95% CI: −0.12, 1.95) (Figure 4b).

(c) Lipid Profile

According to Medina-Vera et al. [28], there were significant reductions in total choesterol, LDL cholesterol, free fatty acids, and triglycerides in the dietary fibre group as compared with the control. Similarly, Reimer et al. [34] noted that the reductions in LDL cholesterol at 16 and 26 weeks in the dietary fibre group were significant as compared with the baseline data. There were significant reductions in total cholesterol and LDL cholesterol [39] in the Ma-Pi 2 diet group as compared with the control, while a study by Soare et al. [41] found no significant differences in relation to total and LDL cholesterol between the two groups.

**Figure 5:**
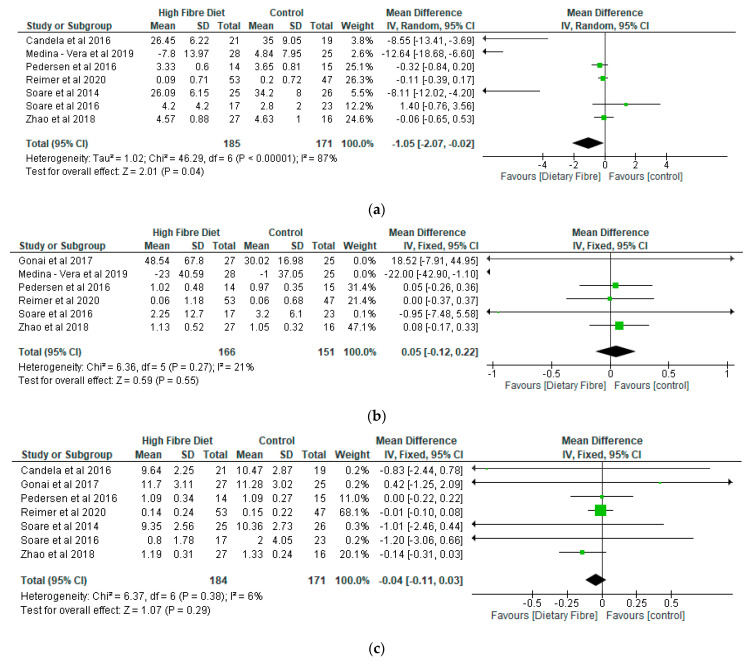

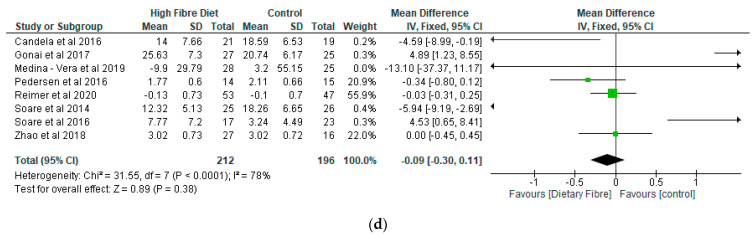
The effect of dietary fibre on (**a**) total cholesterol (mmol/L); (**b**) triglyceride (mmol/L); (**c**) HDL cholesterol (mmol/L); (**d**) LDL cholesterol (mmol/L).

The meta-analyses of the lipid parameters are shown in Figure 5a–d. While there was a decrease of −1.05 (95% CI: −2.07, −0.02) with respect to total cholesterol (356 participants) in the dietary fibre group as compared with the control (*p* < 0.05) (Figure 5a), the two groups were not significantly different (*p* > 0.05) in relation to triglyceride (317 participants), high density lipoprotein cholesterol (355 participants), and LDL cholesterol (408 participants) (*p* > 0.05) (Figure 5b–d).

(d) Inflammatory Markers

**Figure 6:**
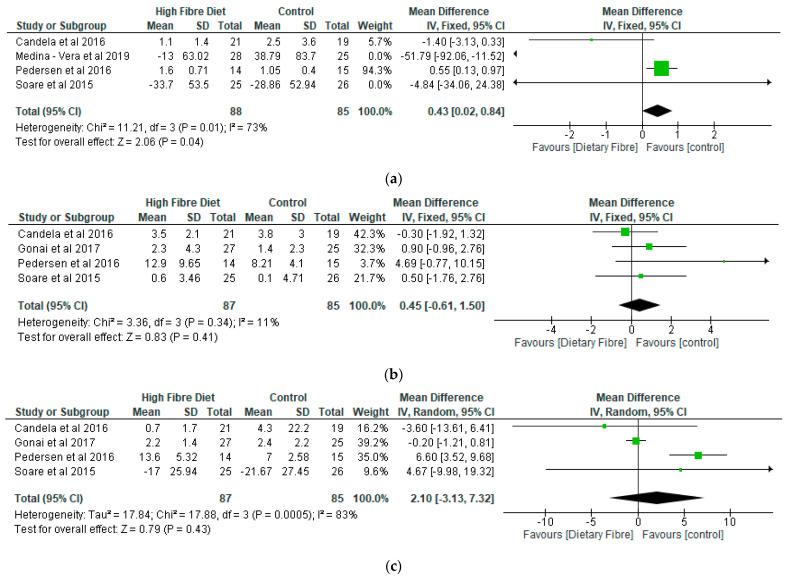

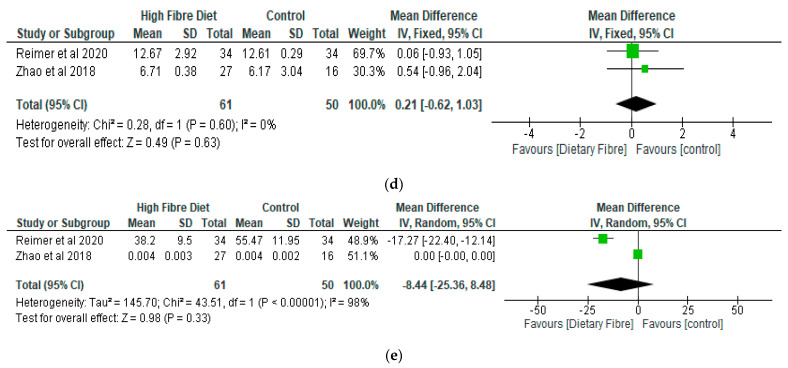
The effect of dietary fibre on (**a**) C-reactive protein (mg/L); (**b**) IL-6 (pg/mL); (**c**) TNF-α (pg/mL); (**d**) adiponectin (μg/mL); (**e**) leptin (ng/mL).

Medina-Vera et al. [28] noted that there were significant reductions in the levels of C-reactive protein in the dietary fibre group as compared with the control. The meta-analysis showed that differences between the two groups was significant (*p* < 0.05) in relation to C-reactive protein, with a mean difference of 0.43 (95% CI: 0.02, 0.84) (Figure 6a). However, there were no significant differences (*p* > 0.05) between the two groups with respect to interleukin 6 (IL-6), tumour necrosis factor α (TNF-α), adiponectin, and leptin (Figure 6b–e).

(e) Body Mass Index (BMI)

Reimer et al. [34] observed that, following the intervention, there was significant decrease in body weight in the dietary fibre group as compared with baseline. In addition, Soare et al. [39] found that BMI in the Ma-Pi diet group was significantly lower than that in the control diet group.

**Figure 7:**
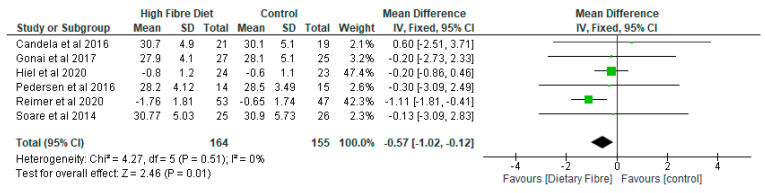
The effect of dietary fibre on body mass index (kg/m^2^).

The meta-analysis of the BMI revealed that the dietary fibre group decreased by −0.57 (95% CI: −1.02, −0.12) as compared with the control group and the difference was significant (*p* < 0.01) (Figure 7).

## 4. Discussion

The results of this systematic review and meta-analysis have shown that dietary fibre was effective in significantly increasing (*p* < 0.05) the relative abundance of *Bifidobacterium*, decreasing lipopolysaccharide, total cholesterol, and body mass index as compared with the control. The dietary fibre group also showed a significant increase in the level of C-reactive protein. However, differences between both groups were not significant (*p* > 0.05) in relation to LBP, triglyceride, HDL cholesterol, LDL cholesterol, IL-6, TNF-α, adiponectin, and leptin.

The findings of this review confirm some results of previous studies and reviews in relation to the role of diet in modulating gut microbiota, lipid profile, and inflammatory markers. They also provide us with a better understanding of the effect of nutritional interventions in managing microbiota-associated diseases such as type 2 diabetes. For example, Haghighatdoost et al. [42] found that the changes in serum levels of IL-6 and TNF-α in the intervention group (resistant starch) as compared with the control group were not significant, which is similar to the current findings. The authors also noted that resistant starch (RS2) that reaches the large intestine in an undigested form may be fermented by gut microbiota and that this can improve the growth of some bacteria families including *Bifidobacteriaceae* and *Lactobacilli*, which can reduce inflammation. Our results align with that observation, as we found that the relative abundance of *Bifidobacterium* significantly increased in the dietary fibre group as compared with the control and that this may have also influenced the significant reduction in the level of LPS in the dietary fibre group.

According to Medina-Vera et al. [28], the reduction in the concentration of LPS by −65% in the intervention group as compared with −52% in the control group showed that dietary interventions could be effective in reducing metabolic endotoxaemia. Gonai et al. [36] noted that, while *Bifidobacteriaceae* was significantly restored in patients with diabetes after consumption of dietary fibre, LBP did not improve during the short-term trial period.

The high level of LPS in the control group may be due to the imbalance of gut microbiota resulting from the lower dietary fibre content [15]. There is evidence that microbial dysbiosis can lead to a loss of integrity of the intestinal wall which could enable the translocation of LPS through the intestinal epithelium, eliciting an inflammatory response and causing oxidative stress, beta cell destruction, and/or insulin resistance [1,4,10]. In addition, high levels of endotoxemia have been shown to increase levels of TNF-α, IL-6, and insulin resistance [12]. According to Haghighatdoost et al. [42], these proinflammatory factors play significant roles in insulin resistance, lipid disorders, and increased oxidative stress.

However, an inflammatory response resulting from LPS is mediated by lipopolysaccharide binding protein (LBP) which is produced mainly in the liver [43,44]. It has been reported that LBP levels increase during infection and are usually greater in the presence of markers of inflammation [43,45]. This would appear to align with the findings of the present review, where there were no significant differences (*p* > 0.05) between the dietary fibre group and the control with respect to LBP and proinflammatory markers, except for C-reactive protein. The significantly higher level of C-reactive protein in the intervention group as compared with the control in the current review may be due to the length of the studies included for C-reactive protein, which were of a relatively short duration (ranging from 21 days to 3 months), and thus not long enough to exert its full effect [36,42]. It could also be due to differences in the participants’ diets including the amount of dietary fibre supplements.

Gut microbiota have been shown to produce short chain fatty acids including propionic, butyric, and acetic acids from the fermentation of the dietary fibre which can lead to improvement in glycometabolism and regulation of the host immune system [6,15,20].

The significantly lower level (*p* < 0.05) of total cholesterol and body mass index found in the dietary fibre group as compared with the control in this review could be ascribed to the effect of the dietary fibre. According to the Scientific Advisory Committee on Nutrition [14], there is sufficient evidence to confirm the association between the compounds that are identified as NSP and colonic function such as stool weight/mass and transit time, and between the compounds that are recognised as soluble fibre and the lowering of total cholesterol and LDL cholesterol. Medina-Vera et al. [28] noted that long-term use of diets high in fibre, rich in polyphenol, and vegetable-protein-based diets could provide beneficial effects in enhancing faecal microbial composition and could provide potential benefits for improving glycamia, dyslipidaemia, and inflammation.

The increased concentration of butyrate, a SCFA, has also been reported to reduce the production of glucose in the liver, improve glucose homeostasis, and reduce body weight [8]. Furthermore, SCFAs have been shown to modulate the metabolism of glucose and lipid through the activation of SCFA receptors on the liver and adipose tissue [8,46]. A weight loss of 5–10% has been found to be adequate in obtaining significant health benefits from decreasing comorbidities [47]. In addition, Wing et al. [48] noted that moderate weight losses from 5 to <10% can improve cardiovascular disease risk factors including glycemia, blood pressure, triglycerides, and HDL cholesterol, but not with respect to LDL cholesterol at 1 year, although it was also recognised that larger weight losses had greater benefits.

### Limitation of the Review

The number of studies that were included in the meta-analysis of gut microbiota and some of the metabolites such as LPS, LBP, and inflammatory markers were limited despite having 10 studies included in the overall meta-analysis. Therefore, the application of the results in the wider context may be limited.

## 5. Conclusions

The findings of this review have shown that dietary fibre can significantly (*p* < 0.05) increase the relative abundance of *Bifidobacterium* and significantly decrease (*p* < 0.05) lipopolysaccharide, total cholesterol, and body mass index as compared with a control. However, the results demonstrated that there were no significant (*p* > 0.05) differences between the dietary fibre group and a control with respect to LBP, triglyceride, HDL cholesterol, LDL cholesterol, IL-6, TNF-α, adiponectin, and leptin. These findings have public health implications in terms of the use of dietary fibre in nutritional interventions and as strategies for managing type 2 diabetes.

## Figures and Tables

**Table 1:** Search terms and search strategy (adapted from Ojo et al. [20]).

Population	Interventions	Outcome	Design of Study	Search Terms Combined
Patients with diabetes	Dietary fibre	Gut microbiota	Randomised controlled trial	
Type 2 diabetes OR Patients with diabetes OR Diabetes OR diabetes mellitus, type 2 OR Diabetes complications OR diabetes mellitus	Fibre OR Dietary fibre OR Supplement OR Prebiotics OR Dietary supplements OR Dietary fibre OR Dietary carbohydrate OR Polysaccharide OR Wheat bran	Microbiome OR Microbiota OR Gastrointestinal microbiota OR Gut microbiota	#1 Controlled clinical trial OR Randomised controlled trial OR randomly OR trial randomised OR placebo OR groups OR drug therapy #2 “Animals” NOT “Humans” #3 #1 NOT #2	Column 1 AND Column 2 AND Column 3 AND Column 4

**Table 2:** The description and characteristics of included studies (adapted from Ojo et al. [20]).

Authors/Country of Study	Type of Study	Details of Sample	Mean Age/Range (Years)	Aim	Type of Interventions	Findings
Birkeland et al. [37], Norway	RCT	n = 25	63.1: 41–73	To examine the effect of inulin-type fructans on faecal microbiota and short chain fatty acids in patients with type 2 diabetes.	Inulin-type fructans (a mixture of oligofructose and inulin) versus placebo (maltodextrin) A 4 week washout separated 6 weeks of treatment	The results found a moderate potential of inulin-type fructans to promote the composition of gut microbiota and to increase microbial fermentation in T2D.
Candela et al. [38], Italy	RCT	Ma-Pi 2 diet (n = 21), control diet (n = 19)	66	Two different energy-restricted dietary approaches were explored, i.e., the fibre-rich macrobiotic Ma-Pi 2 diet or a control diet	Macrobiotic Ma-Pi 2 diet rich in fibre versus control diet. A 21-day treatment	The Ma-Pi 2 diet was effective in alleviating the increase of possible proinflammatory groups, in the gut ecosystem, but not the control diet. It demonstrated the possibility of reversing proinflammatory dysbiosis in patients with T2D and its effectiveness in improving metabolic control.
Gonai et al. [36], Japan	RCT	GOS (n = 27), placebo (n = 25)	GOS (55 ± 11) Placebo (54 ± 12)	To evaluate the role of GOS on glycaemic control, gut microbiota, and metabolites in patients with type 2 diabetes.	GOS versus placebo (maltodextrin) A 4-week treatment	*Bifidobacteriaceae* was significantly restored in patients with diabetes after consuming GOS. On the other hand, there was no improvement in LBP and glucose tolerance during this short period of trial. It was shown that GOS could mitigate dysbiosis in patients with diabetes, and continuous intake of GOS may be useful in managing type 2 diabetes.
Hiel et al. [33], Belgium	RCT	47 Metformin-treated participants (all diabetic, prebiotic n = 24, placebo n = 23)	Age ranged from 18 to 65 years.	To explore the effect of inulin supplementation with metformin in obese patients with T2D and their beneficial effects through modulation of gut microbiota.	Subjects were randomly assigned to the prebiotic or placebo arm A 3-month treatment	A large increase in *Bifidobacterium* may be due to inulin intake rather than a driver of prebiotic-linked biological outcomes.
Medina-Vera et al. [28], Mexico	RCT	T2D (n = 81) Final Group numbers analysed: DF (n = 28), placebo (n = 25)	DP (50.4 ± 8.7) Placebo (49.8 ± 10.6)	To examine the role of dietary intervention (functional food-based) on faecal microbiota and biochemical parameters in patients with type 2 diabetes.	A dietary portfolio (DP) versus placebo A 3-month treatment	The long term use of diets that are high in fibre, rich in polyphenol and vegetable-protein-based provide advantages in enhancing the faecal microbiota composition and may be used as therapies for managing dyslipidaemia and inflammation.
Pedersen et al. [29], UK	RCT	GOS (n = 14), placebo (n = 15)	GOS (56.7 ± 1.6) Placebo (58.1 ± 1.7)	To compare the effects of prebiotic supplementation with placebo treatment in patients with type 2 diabetes.	GOS versus placebo (maltodextrin) A 12-week treatment	As compared with the placebo, supplementation with prebiotic fibre did not appear to show any significant impact on clinical outcomes or bacterial abundances.
Reimer et al. [34], Canada	RCT	PGX^®^ (n = 147), placebo (n = 143)	PGX^®^ (56.2 ± 8.6) Placebo (53.4 ± 9.9)	To evaluate the adjunct effect of the soluble viscous fibre PGX^®^ on glycemic control in patients with T2D.	PGX^®^ versus placebo A 52-week treatment.	PGX^®^ may be a useful adjunct to weight loss programs in patients with type 2 diabetes based on improvements in other metabolic parameters.
Soare et al. [39], Italy	RCT	Ma-Pi 2 diet (n = 25), control diet (n = 26)	Ma-Pi 2 diet (67 ± 8.163) Control diet (65 ± 7.284)	The effect of various dietary methods (the macrobiotic Ma-Pi 2 diet) were compared with standard diets recommended for patients with type 2 diabetes.	Fibre-rich macrobiotic Ma-Pi 2 diet versus control diet A 21-day treatment	There was significantly better improvements in metabolic control in patients with type 2 diabetes following the intervention with a short-term Ma-Pi 2 diet.
Soare et al. [40], Italy	RCT	Ma-Pi 2 diet (n = 25), control diet (n = 26)	Age ranged from 40 to 75 years	To investigate the effects of macrobiotic Ma-Pi 2 diet versus a standard recommended diet (control diet) on inflammatory markers in patients with T2D.	This was a post hoc analysis of the MADIAB trial A 21-day RCT.	As compared with the baseline data, it was found that Ma-Pi 2 diet was a safe dietary method of reducing levels of inflammatory markers, in the short term.
Soare et al. [41], Italy	RCT	Ma-Pi 2 diet (n = 17), control diet (n = 23)	Ma-Pi 2 diet (65 ± 8.89) Control diet (64 ± 8.15)	Evaluation of the advantages of the original 21-day intensive dietary interventions beyond the original MADIAB trial duration and into everyday life.	Fibre-rich macrobiotic Ma-Pi 2 diet versus control diet A 6-month follow-up study	There was higher percentage reduction in body weight and a higher percentage increase in LDL cholesterol in the Ma-Pi diet. Furthermore, all the participants’ total and LDL cholesterol levels were within recommended levels.
Zhao et al. [35], China	RCT	High dietary fibre (n = 27), control (n =16)	High dietary fibre (58.4 ± 6.2) Control (59.7 ± 6.0)	To assess the effect of gut microbiota and its role in glucose homeostasis in patients with type 2 diabetes.	High dietary fibre versus usual care A 84 days study	Dietary fibre was effective in promoting a group of SCFA-producing strains, while most of the other potential producers were either reduced or unchanged in patients with type 2 diabetes.

Abbreviations: DP (dietary portfolio); GOS (galacto-oligosaccharide); LBP (lipopolysaccharide binding protein); LDL (low density lipoprotein) cholesterol; Ma-Pi 2 (macrobiotic diet); PGX^®^ (PolyGlycopleX^®^); SCFA (short chain fatty acid); T2D (type 2 diabetes); RCT (randomised Controlled Trial).
